# The Cognitive Control of Memory: Age Differences in the Neural Correlates of Successful Remembering and Intentional Forgetting

**DOI:** 10.1371/journal.pone.0087010

**Published:** 2014-01-24

**Authors:** Avery A. Rizio, Nancy A. Dennis

**Affiliations:** The Pennsylvania State University, Department of Psychology, State College, University Park, Pennsylvania, United States of America; Emory University, United States of America

## Abstract

Successful memory encoding depends on the ability to intentionally encode relevant information (via differential encoding) and intentionally forget that which is irrelevant (via inhibition). Both cognitive processes have been shown to decline in aging and are theorized to underlie age-related deficits in the cognitive control of memory. The current study uses the Directed Forgetting paradigm in conjunction with fMRI to investigate age-related differences in both cognitive processes, with the specific aim of elucidating neural evidence supporting these theorized deficits. Results indicate relatively preserved differential encoding, with age differences consistent with previous models of age-related compensation (i.e., increased frontal and bilateral recruitment). Older adults did display noticeable differences in the recruitment of brain regions related to intentional forgetting, specifically exhibiting reduced activity in the right superior prefrontal cortex, a region shown to be critical to inhibitory processing. However, older adults exhibited increased reliance on processing in right inferior parietal lobe associated with successful forgetting. Activity in this region was negatively correlated with activity in the medial temporal lobe, suggesting a shift in the locus of inhibition compared to the frontally mediated inhibition observed in younger adults. Finally, while previous studies found intentional and incidental forgetting to be dissociable in younger adults, this differentiation appears to be reduced in older adults. The current results are the first to provide neural evidence for an age-related reduction in processes that support intentional forgetting.

## Introduction

Do older adults have difficulty forgetting? At first glance this may appear to be a nonsensical question, especially when considering the vast literature documenting age-related memory impairments [2], [3], [4]. However, forgetting is not a unitary function. While the oft-investigated incidental forgetting may simply be a by-product of poor encoding operations, thereby impairing successful encoding, intentional or goal-directed forgetting is considered to be a strategic process, benefitting memory by reducing interference associated with the processing of irrelevant information. This latter form of forgetting falls under the realm of control operations that influence memory performance. While the majority of behavioral evidence shows an increased rate of incidental forgetting in older adults, the ability to engage cognitive control processes to support intentional forgetting has also been shown to diminish with age [1], [5], [6]. While a handful of studies have investigated the neural mechanisms underlying memory deficits resulting from age-related increases in incidental forgetting [7], the neural basis underlying age deficits in intentional forgetting has yet to be examined. The current study seeks to fill this gap in the literature by elucidating age-related differences in the neural correlates mediating both encoding and inhibition as they pertain to intentional forgetting.

With regard to memory encoding, cognitive control processes allow for successful encoding/storage of relevant information, while also suppressing the encoding of irrelevant information. In the lab, such control processes have been studied using the Directed Forgetting (DF) paradigm. In item-method DF participants are presented with a series of items (e.g., words), each followed by an instruction to either remember or forget. It is theorized that until the memory cue is presented, the item is held in working memory [8], [9]. When the memory cue is presented, participants either engage encoding processes aimed at remembering the item (in the case of a remember cue) or engage inhibitory processes aimed at suppressing encoding and inducing forgetting (in the case of a forget cue). While memory for all items is tested in a subsequent memory test, control over memory is measured with respect to the proportion of items subsequently remembered that were originally associated with a to-be-remembered (TBR) cue opposed to a to-be-forgotten (TBF) cue. Research has posited that individuals achieve control over memory encoding in the DF paradigm via two separable mechanisms: differential encoding and attentional inhibition [4], [10], [11], [12], [13]. DF studies find that while both younger and older adults exhibit control over memory encoding (remembering more TBR than TBF items), the magnitude of this effect is reduced in older adults [1], [14], [15], [16]. Age deficits in both differential encoding and attentional inhibition are believed to contribute to the behavioral deficit in DF [1], [4].

Differential encoding engages deep, elaborate encoding processes for TBR items, yet not TBF items [8], [17]. DF studies using event-related potentials (ERPs) find that differential encoding is associated with greater positivity in posterior cortices for TBR as compared to TBF cues [9], [18]. FMRI studies have extended this work to show that the presentation of a cue to remember, compared to a cue to forget, activates regions of the left inferior prefrontal cortex, cingulate gyrus, and occipital cortex [10], [13], [19], [20]. In addition, successful intentional remembering in the DF paradigm (intentional remembering compared to intentional forgetting) is associated with increased activity in the medial temporal lobe (MTL), striatum, left superior and inferior temporal gyri, and left parietal cortex [10], [13]. Together, results support the theory that increased and more elaborate encoding processes support intentional remembering.

While no DF study has examined the neural basis of differential encoding in older adults, episodic encoding studies find that while both younger and older adults exhibit encoding activity across a number of brain regions including the left dorsolateral and ventrolateral prefrontal cortex (PFC), MTL, and occipital cortex, older adults exhibit reductions in encoding activity in posterior brain regions, while exhibiting increased activity throughout the PFC [21], [22], [23], [24], [25], [26], [27], [28]. Decreases in posterior activations are reflective of age-related changes in the recruitment of regions related to perception necessary for successful memory performance, while increased PFC activity may be recruited to offset these posterior reductions [24], [29], [30], [31], [32], [33]. While the foregoing findings are based largely on incidental encoding tasks, the current study provides the opportunity to investigate age-related changes in the neural correlates of self-initiated encoding success. We expect that older adults will exhibit reductions in both self-initiated processing and neural activity related to encoding attempt. Specifically, we predict age-related reductions in the strength of differential encoding activity localized to posterior regions including occipital cortex and the MTL. In accord with previous results from incidental encoding tasks, we also expect age-related increases in engagement of frontal activity.

While differential encoding enhances encoding processes for relevant information (i.e., TBR items), attentional inhibition inhibits processing of irrelevant information (i.e. TBF items). By suppressing encoding, information is prevented from entering long term memory [1], [9], [10], [34]. Given the role of the superior and middle frontal gyri in cognitive control and inhibition tasks in related domains (e.g. retrieval suppression) [35], [36], [37], [38], [39], DF studies have focused on the role of the right prefrontal cortex as the locus of inhibitory control. For example, ERP studies find differential activation of the right PFC for TBF as opposed to TBR cues in the DF paradigm [9], [34], [40], while fMRI studies find increased right superior PFC activity for TBF compared to TBR instructions [10], [13], [20]. Supporting the role of the right PFC in inhibitory processes, functional connectivity analyses have found activity in the right superior and middle PFC to be negatively correlated with memory-related processing in the MTL concurrent with forgetting instructions [13], [35], [37], [41]. In addition to the right PFC, inferior parietal cortex activity has also been found to support intentional forgetting in DF [10], [13], [34]. The role of parietal cortex in supporting memory control has been studied recently in younger adults, but its contribution has not yet been studied with respect to aging.

While critical to successful performance in the DF paradigm, inhibition has been shown to undergo age-related decline across a wide range of cognitive tasks, including the ability to ignore irrelevant text in reading comprehension tasks and the ability to suppress outdated information during memory-related retrieval tasks [4], [5], [42], [43], [44], [45], [46]. Neuroimaging studies have associated this behavioral change with decreased right frontal activation [47], [48], [49] as well as increased left frontal activity, illustrating the ways in which task-related networks may adapt over the lifespan [49], [50]. Connectivity research in younger adults has demonstrated that the PFC suppresses encoding and retrieval related activity in the MTL during memory inhibition tasks [13], [35], [37], [41]. However, if older adults are unable to recruit the PFC to the same extent as younger adults, it is likely that this region will be less able to suppress encoding-related activity. Taken together, previous results strongly suggest that older adults’ behavioral impairments in information selection and inhibition are associated with age-related differences in neural activations, particularly with respect to activity in the PFC and connectivity between this region and the MTL.

Though the aforementioned findings suggest that older adults experience a decline in the ability to inhibit encoding of irrelevant information, no study to date has examined the neural basis of inhibition processes in the DF paradigm in aging. Given the role of the right superior and middle PFC in cognitive inhibition we expect that this region will be essential to the completion of successful intentional forgetting in older adults, and age-related deficits in DF will be associated with reduced recruitment of right PFC activity and reduced connectivity with MTL during intentional forgetting. Moreover, as older adults’ ability to successfully recruit regions that support intentional forgetting decreases, the dissociation between intentional and incidental forgetting should also diminish.

## Method

### Ethics Statement

The study was approved by the Pennsylvania State University Institutional Review Board. All participants provided written informed consent.

### Participants

Twenty-seven younger adults between the ages of 18–24 (*M* = 20.92, *SD* = 1.61) and 27 older adults between the ages of 61–84 (*M* = 71.00, *SD* = 6.68) participated for monetary compensation. The data of two younger participants were lost due to scanner malfunction; data from 1 young and 3 older adults were excluded due to the participants’ failure to follow instructions. Additionally, one older adult was excluded because an incidental finding was observed during scanning. Thus, 24 younger adults and 23 older adults were included in the final analysis. All participants were healthy, right-handed, native English speakers, with no history of neurological or psychiatric episodes. Before scanning all participants completed a battery of neuropsychology tests comprised of the Mini-Mental State Examination, the Beck Depression Inventory, and sections of the Wechsler Adults Intelligence Scale (WAIS, version III, see Table 1). All participants scored above 27 on the MMSE and no participant scored below two standard deviations of the age-matched normative score on any of the other cognitive tests (see table 1 for group means and standard deviations). All participants provided written informed consent, and the Pennsylvania State University Institutional Review Board approved all procedures.

**Table 1:** Participant demographic and neuropsychological testing information.

	Younger Adults	Older Adults
	Mean (SD)	Mean (SD)
**Demographic Information**		
N	24	23
Age*	20.92 (1.61)	71 (6.68)
Gender (M/F)	8/16	6/17
Education (Years)*	14.43 (1.24)	17.30 (2.60)
**Neuropsychological Testing**		
MMSE	29.71 (0.46)	29.70 (0.63)
Symbol Search*	41.50 (6.63)	31.48 (4.79)
Symbol Copy*	129.96 (8.82)	113.14 (15.96)
Digit Symbol Coding*	87.71 (12.23)	70.30 (13.29)
Digit Span	19.13 (4.48)	17.65 (2.89)
Arithmetic	13.13 (4.15)	13.74 (3.36)
Letter-Number Sequencing*	11.92 (2.57)	10.00 (2.54)
Vocabulary	53.38 (6.85)	55.61 (4.57)
Beck’s Depression Inventory	2.50 (2.80)	04.52 (4.02)

* Scores for which a significant age difference exists, *p*<0.05.

## Materials

Three hundred sixty nouns were chosen from the MRC Psycholinguistic Database. Words had an average Kucera-Francis written frequency of 110 (range: 50–275), and an average concreteness of 433 (range: 254–600). One hundred words were randomly marked as to-be-remembered (TBR) and another 100 as to-be-forgotten (TBF) during encoding, and the remaining 160 were used as “new” items during the recognition test.

### Procedure

**Figure 1:**
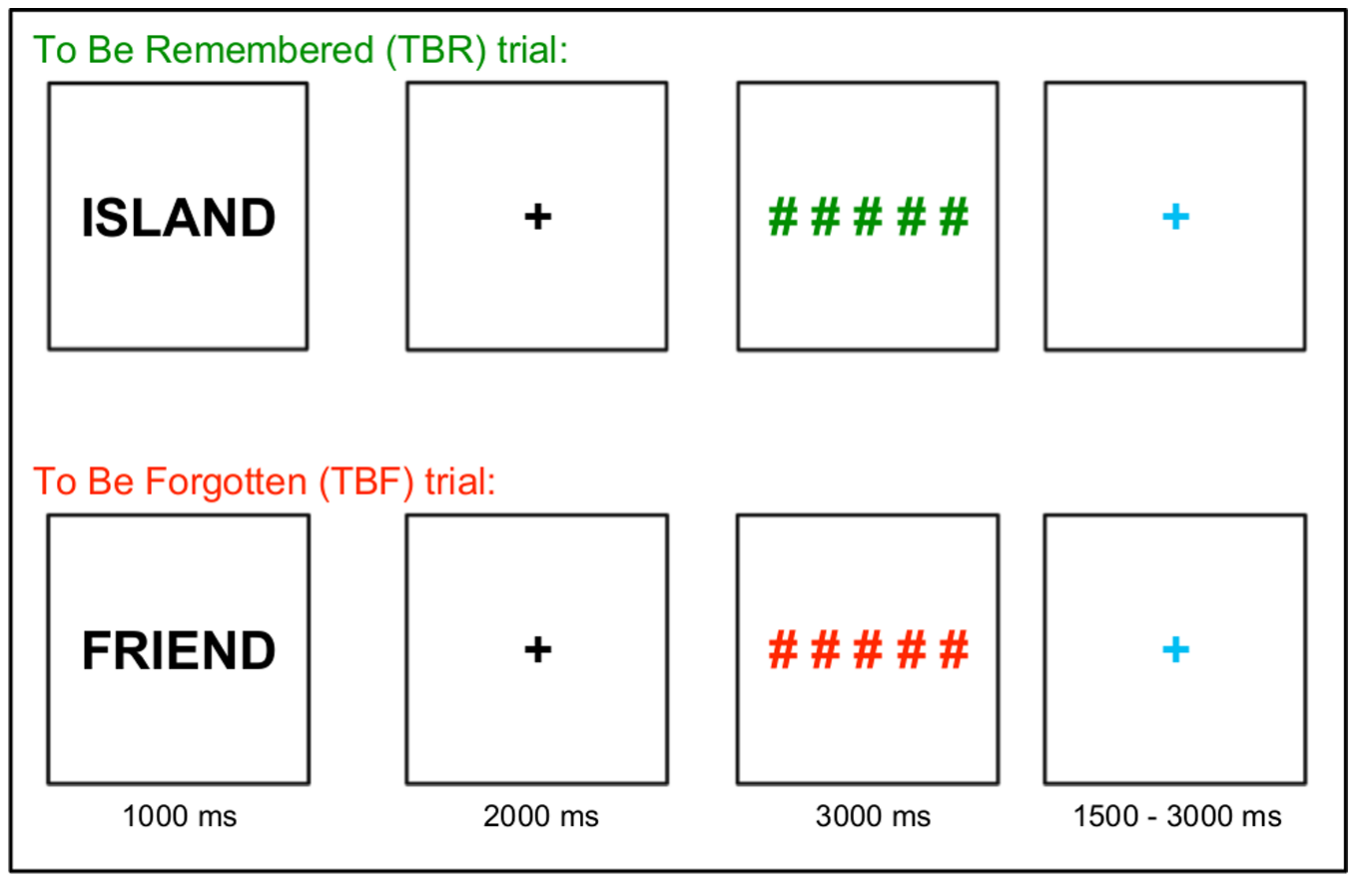
Item method directed forgetting encoding task. Participants were instructed that words associated with green pound/number signs should be remembered for an upcoming memory test, while words associated with red pound/number signs should be forgotten, because they would not be on the test.

The experiment employed a traditional item method DF paradigm. During encoding, each of the 200 words appeared individually on the screen for 1000 ms, and was followed by a fixation cross that remained on the screen for 2000 ms. Following the delay, a set of five colored number signs was presented for 3000 ms (see Figure 1). Participants were instructed that words followed by green number signs were to-be-remembered (TBR), as they would appear on an upcoming memory test, and words followed by red number signs were to-be-forgotten (TBF), as they would not be on the memory test (see Figure 1). The encoding trials were broken into five blocks of 40 words, with TBR and TBF items appearing in a pseudorandom order, such that no more than three of the same trial type appeared sequentially. Each trial was followed by a jittered fixation that lasted between 1500 and 3000 ms, and averaged 2000 ms.

Following encoding, and prior to retrieval, participants completed a 10-minute interference task (the matrix reasoning subtest of the WAIS). Next, participants performed a retrieval task, which included a total of 360 words: the 100 TBR items and 100 TBF items from encoding, and 160 new words. Each word appeared individually on the screen for 2500 ms, and participants made a Remember/Know/New memory decision during that time [51]. It was stressed to the participants that their memory response should *not* depend on whether the word had been marked as TBR or TBF during the study phase, but should instead depend only on whether the word was old or new. Retrieval was also scanned, but not included in the current analysis.

### Image Acquisition

Imaging data were acquired using a 3 T Siemens Magnetom Trio MRI scanner. Functional encoding data were obtained in five, 5.60-minute runs, each consisting of a total of 165 volumes. Images were collected using an echo-planar imaging (EPI) sequence with a two-second repetition time (TR), 30 ms echo time (TE), 240 mm field of view (FOV), and a 70 degree flip angle. Thirty-four slices were acquired per TR, with a slice thickness of 3.8 mm, resulting in 3.8 mm^3^ isotropic voxels. Structural images were acquired during the interference task, using a T1 weighted gradient echo sequence MPRAGE, with a TR of 2300 ms, a TE of 3.41 ms, a 230 mm FOV, and a voxel size of 0.9 mm^3^.

### Image Processing

Preprocessing and statistical analyses were performed using Statistical Parameter Mapping software in MATLAB (SPM 8; Wellcome Department of Cognitive Neurology, London, UK). First, time-series data were corrected for differences in slice acquisition time. Images were then spatially realigned to the first functional run of encoding and were subsequently checked for movement artifacts using a time series diagnostic function TSDiffAna (Freiburg Brain Imaging) in MATLAB (MathWorks). No individual moved more than 3 mm in any direction, in any run and thus no data were removed due to motion artifacts. The functional images were then normalized using the Montreal Neurological Institute (MNI) template. Clusters of significant activation were then converted to Talairach coordinates [52] and reported as such in all tables. Finally, images were smoothed using an 8 mm Gaussian smoothing kernel.

### Data Analysis

Trial-related activity was modeled with a general linear model (GLM) for each participant with a stick function corresponding to stimulus onsets, convolved with a hemodynamic response function (HRF), in accord with our previous analysis of the young data [13]. Confounding factors including head motion and magnetic field drift were also included in the model. Statistical Parametric Maps were identified for each participant by applying linear contrasts with the parameter estimates (beta weights) for the events of interest, resulting in a *t-*statistic for every voxel.

Given that our study goal was to examine neural activity associated with the execution of control processes in memory and age differences therein, we focused our analysis on neural activity associated with the onset of memory cues (TBF/TBR). As noted above, it is assumed that activity associated with the word is equitable across trials and differential processing associated with memory control occurs in response to the presence of the memory instruction. As such, and in accord with previous DF studies [10], [13], [53], word-related activity was modeled, but treated as a regressor of no interest in the analyses.

Encoding trials were coded using a subsequent memory design. Given memory instructions (TBR/TBF) and the subsequent memory scoring (recollection/familiarity/forgotten), the model included six trial types of interest: (1) TBR-Recollection: intentional remembering; (2) TBR-Forgotten: incidental forgetting; (3) TBF-Recollection: incidental remembering; and (4) TBF-Forgotten: intentional forgetting. Subsequent familiarity [(5) TBR-Familiarity and (6) TBF-Familiarity] were also modeled and included in analyses examining encoding and forgetting attempt The choice to focus on recollection was based on our interest in assessing the role of cognitive control and inhibition processes as they pertain to the most item-specific form of recognition memory. Additionally, because recollection and familiarity have been shown to rely on distinct regions of the medial temporal lobe, focusing entirely on recollection ensures that the memory signal is not weakened by the inclusion of two separate, independent memory response [54], [55].

Encoding attempt activity was defined as activity associated with all TBR trials compared to TBF trials. Encoding success was defined by comparing activity associated with TBR-Recollection with TBF-Forget. Likewise, forgetting attempt was defined as activity associated with all TBF trials compared to TBR trials, and forgetting success was defined by comparing activity associated with TBF-Forget trials with TBR-Recollection trials. Lastly, the dissociation between intentional forgetting and incidental forgetting was examined by comparing activity between TBF-Forget and TBR-Forget. The foregoing contrasts were chosen in order to best mirror those used previously to operationalize encoding and inhibition attempt as well as success in the context of both Directed Forgetting and Think/No-Think paradigms [10], [13], [37], [38], [40], [41]. Using a similar methodology across tasks helps to promote consistency across the few studies that use neuroimaging to investigate intentional forgetting, thus allowing for ease of comparison across published results.

In order to obtain results that were corrected for multiple comparisons we used Monte Carlo simulations (http://www2.bc.edu/slotnics/scripts.htm) to define individual voxel and cluster extent thresholds [56], [57], [58], [59], [60]. For each contrast of interest, an individual voxel threshold of p<0.005 was used in combination with a cluster extent threshold of 19 resampled voxels (1043 mm^3^), which yielded whole-brain results corrected for multiple comparisons at *p*<0.05.

Age differences were assessed for each contrast of interest using a region-of-interest (ROI) approach, such that group differences were computed only within regions that exhibited significant activation within each age group. For example, in order to identify regions where younger adults exhibited greater activation than older adults related to successful intentional forgetting the following procedure was employed: Successful intentional forgetting was assessed within younger adults at the aforementioned corrected threshold of *p*<0.05. The results were then used as an inclusive mask for assessing age differences at *p*<0.05 uncorrected. As a final step, we further guarded against false positives in the age difference analysis by employing a similar extent threshold correction to the results. Specifically, using both the uncorrected p-value of.05 and the area within the ROI mask, we obtained a corrected cluster threshold. (In all cases this correction resulted in 17 contiguous voxels). A similar procedure was then used to assess regions where older adults exhibited greater activation than younger adults for successful intentional forgetting, as well as for all other contrasts of interest.

By employing a corrected threshold for the individual group data and using it as an inclusive mask for corrected between group contrasts we were able to focus our age difference analyses only within regions that were of primary significance to either the young or older adults. This procedure also ensures that age differences were driven by increases in activation in the primary group of interest (e.g., young adults in a young>old comparison) rather than deactivations in the subsequent group (e.g., older adults). Additionally, we are able to conclude that regions identified with this analysis approach were both significantly activated in one group and, through a focal analysis on that region, that the region exhibited a significant group difference [23], [61], [62], [63].

### Connectivity Analysis

To investigate whether activity associated with intentional forgetting reflects inhibition of encoding processes, we conducted a PPI (psychophysiological interaction) analysis. The seed voxel for the PPI analysis was chosen from the subset of regions found to be significantly active for successful intentional forgetting in older adults. While we anticipated investigating age-related reductions in frontally-mediated inhibition, no region in right PFC was found to be significantly active in older adults in our inhibition contrast. As such we chose to conduct the connectivity analysis on a region in right parietal cortex that, like young adults [13], also showed significant inhibition-related activity. Specifically, we used the peak voxel from an activated cluster in the right inferior parietal lobe (48, −44, 22; see table S2 and figure S1). Choice of this seed was based on both previous evidence implicating the right parietal lobe in inhibitory processing and the identification of this region as the location of successful intentional forgetting in older adults in the current study. A sphere with a radius of 4 mm was drawn around the peak, and the time course of TBF-Forget trials was extracted for the resulting seed region. The time course was then compared with the time courses of voxels within the MTL, using a mask of the MTL and a threshold of *p*<0.05, corrected (which is equal to *p*<0.05 uncorrected, and 13 contiguous voxels within the MTL ROI).

The MTL ROI was defined anatomically through the SPM 8 pickatlas, and included the left and right hippocampi and parahippocampal gyri. We focused our PPI analysis within the MTL because both previous evidence and our current study have implicated the MTL in mediating encoding success. The same time course extraction procedure was also conducted for TBR-Recollection trials, TBF-Recollection trials and TBR-Forget trials.

While it would be ideal to analyze connectivity from a common seed region across both age groups, this method would have introduced a clear bias in the results, as younger and older adults did not exhibit any exact overlap in parietal or prefrontal regions. Given the constraints imposed by our data, our choice of methodology represents the best alternative to using common seed regions.

## Results

### Behavioral Results

[Young adult data have been previously reported [13] and are repeated here for comparison purposes.].

Traditionally, the DF paradigm includes a yes/no recognition test, which collapses across recollection and familiarity. The presence of only two response options means that remembering and forgetting scores are inverses of each other, and thus allows for only one measure of memory control, the DF effect. The current DF paradigm, however, separates both recollection and familiarity from forgetting, thus requiring two measures of memory control: one that measures directed remembering, and one that measures directed forgetting. Both younger and older adults exhibited a significant remembering effect (younger: *t*(23) = 5.57, *p*<0.001; older: *t*(22) = 5.11, *p*<0.001), such that both age groups exhibited a greater rate of intentional remembering (TBR-Recollection: younger: *M* = 0.42, *SE = 0.04*; older: *M* = 0.45, *SE* = 0.03) than incidental remembering (TBF-Recollection: younger: *M* = 0.26, *SE* = 0.03; older: *M* = 0.34, *SE* = 0.03).

Both younger and older adults also exhibited a significant forgetting effect (younger: *t*(23) = 5.85, *p*<0.001; older: *t*(22) = 3.54, *p*<0.005), such that both age groups exhibited a greater rate of intentional forgetting (TBF-Forget: younger: *M* = 0.39, *SE* = 0.03; older: *M* = 0.35, *SE* = 0.04) than incidental forgetting (TBR-Forget: younger: *M* = 0.26, *SE* = 0.02; older: *M* = 0.27, *SE* = 0.03).

While both the remembering effect and forgetting effect were numerically larger in younger compared to older adults, a comparison between age groups revealed that there was no significant age difference between either memory effect, [remembering-effect: *t*(46) = 1.78, *ns*; forgetting effect *t*(46) = 1.55, *ns*]. Similar results were obtained using the traditional DF effect as well (collapsing across recollection and familiarity), *t* (46) = 0.11. *ns*.

Although age differences in directed forgetting and remembering effects were not significant, other behavioral differences did emerge. Specifically, there was a significant age difference in the rate of incidental remembering, such that older adults (*M* = 0.34, *SE* = 0.03) recollected significantly more TBF items than younger adults (*M* = 0.26, *SE = *0.03), *t*(46) = 2.12, *p*<0.05. Additionally, driven by age differences in false alarms, there was a significant difference in d-prime scores, such that younger adults (*M* = 1.34, *SE* = 0.12) were more sensitive to detecting the difference between targets and lures than older adults (*M* = 0.92, *SE* = 0.11), *t*(46) = 2.50, *p*<0.05. (see Table 2 for complete behavioral results).

**Table 2:** Rate of response as a function of trial type and age.

Word Type	Younger Adults	Older Adults
	Mean (SE)	Mean (SE)
**TBR**		
Recollection	0.43 (0.04)	0.45 (0.03)
Familiarity	0.30 (0.02)	0.27 (0.03)
Forget	0.26 (0.02)	0.27 (0.03)
**TBF**		
Recollection*	0.26 (0.03)	0.34 (0.03)
Familiarity	0.35 (0.02)	0.30 (0.03)
Forget	0.39 (0.03)	0.35 (0.04)
**Directed Forgetting Effects**		
Remembering Effect	0.18 (0.03)	0.11 (0.02)
Forgetting Effect	0.12 (0.02)	0.08 (0.02)
**Foil**		
False Recollection*	0.11 (0.03)	0.25 (0.03)
False Familiarity	0.34 (0.03)	0.38 (0.05)
Correct Rejection	0.66 (0.05)	0.80 (0.10)
**Sensitivity (d’)** *	1.34 (0.12)	0.92 (0.11)

TBR = To-be-remembered; TBF = To-be-forgotten; SE = Standard Error.

Remembering Effect = TBR-Recollection – TBF-Recollection.

Forgetting Effect = TBF-Forget – TBR-Forget.

* Scores for which a significant age difference exists, *p*<0.05.

### Neuroimaging Results

As the focus of the current investigation was to elucidate neural correlates mediating age-related differences in both intentional remembering and intentional forgetting only age differences are presented below. Results from younger adults were presented in a previous study [13] and results from older adults are presented in Table S1, Table S2 and Table S3.

### Encoding Attempt (TBR>TBF) and Encoding Success (TBR-R>TBF-F)

#### Younger adults>Older adults

**Figure 2:**
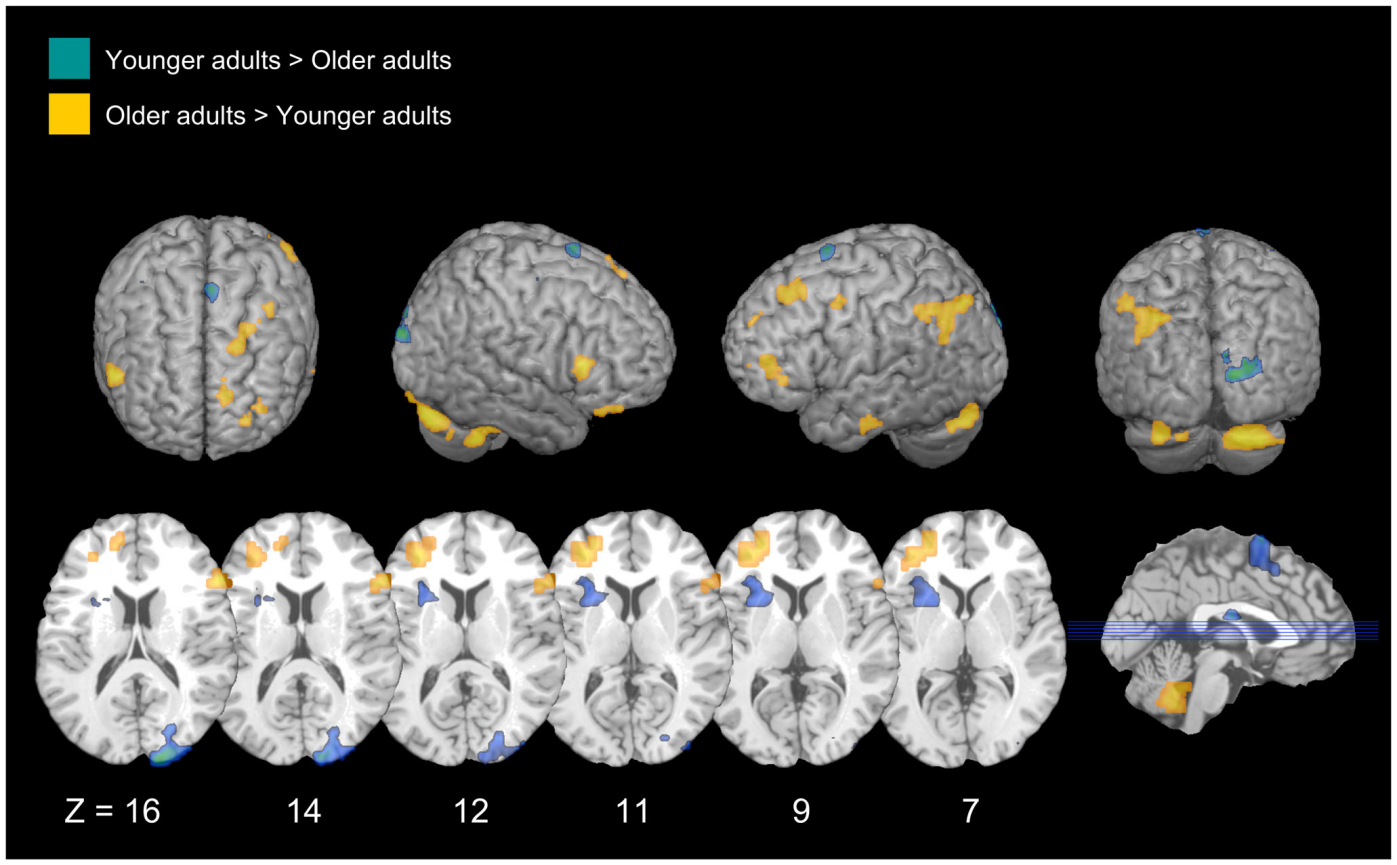
Age differences in successful intentional remembering. Areas that are significantly more active for successful intentional remembering than successful intentional forgetting, and for which that difference is either greater in younger adults than older adults (blue), or greater in older adults than younger adults (yellow).

In line with our predictions, younger adults exhibited increased neural activity in the occipital cortex during encoding attempt, as compared to older adults. During successful encoding, younger adults exhibited increased neural activity in the prefrontal and occipital cortices, as compared to older adults (see Table 3 & Figure 2).

**Table 3:** Intentional Remembering.

	BA	H	Coordinates (T&T)			*t*	mm^3^
			*X*	*Y*	*Z*		
**Remembering Attempt: TBR>TBF**							
**Younger Adults>Older Adults**							
Middle Occipital Gyrus	18	R	22	−100	18	2.90	1317
**Older Adults>Younger Adults**							
Middle Frontal Gyrus	11	L	−11	48	−16	2.24	1207
Superior Frontal Gyrus	6	L/M	−7	−25	59	3.00	1207
Precentral Gyrus/Frontal Eye Fields	6	R	63	−18	42	4.26	3896
	6, 4, 3	L	−48	−12	24	3.33	4774
Inferior Parietal Lobe	40	L	−48	−43	57	2.52	1317
Inferior Temporal Gyrus	37	L	−56	−57	−14	2.38	1427
**Successful Remembering: TBR-R>TBF-F**							
**Younger Adults>Older Adults**							
Superior Frontal Gyrus	6	M	−4	12	68	3.44	5158
	6	R	41	−3	62	2.71	1207
Putamen	–	L	−26	13	6	2.53	9657
Early Visual Cortex	18	R	15	−95	28	4.39	6146
	18	R	7	−71	0	2.63	1317
**Older Adults>Younger Adults**							
Middle Frontal Gyrus	10	L	−30	46	7	3.09	2634
	10	L	−30	7	37	2.44	1262
	11	R	22	34	−9	2.52	1482
Inferior Frontal Gyrus	45	R	59	28	15	3.75	1097
Superior Frontal Gyrus	8	L	−22	37	46	2.63	2140
Middle Temporal Gyrus/Angular Gyrus	39	L	−37	−56	26	3.41	5981

BA = Brodmann’s Area; H = Hemisphere; *t* = statistical *t* value; T&T = Talairach and Tournix coordinates.

R = Right; L = Left; M = Midline.

#### Older adults>Younger adults

Encoding attempt was associated with greater activity in regions of the prefrontal, frontal, parietal, and temporal cortices for older adults compared to younger adults. With respect to successful intentional encoding, older adults exhibited increased neural activity in the bilateral prefrontal and left temporal cortices (see Table 3 & Figure 2).

### Forgetting Attempt (TBF>TBR ) and Forgetting Success (TBF-F>TBR-R)

#### Younger adults>Older adults

**Figure 3:**
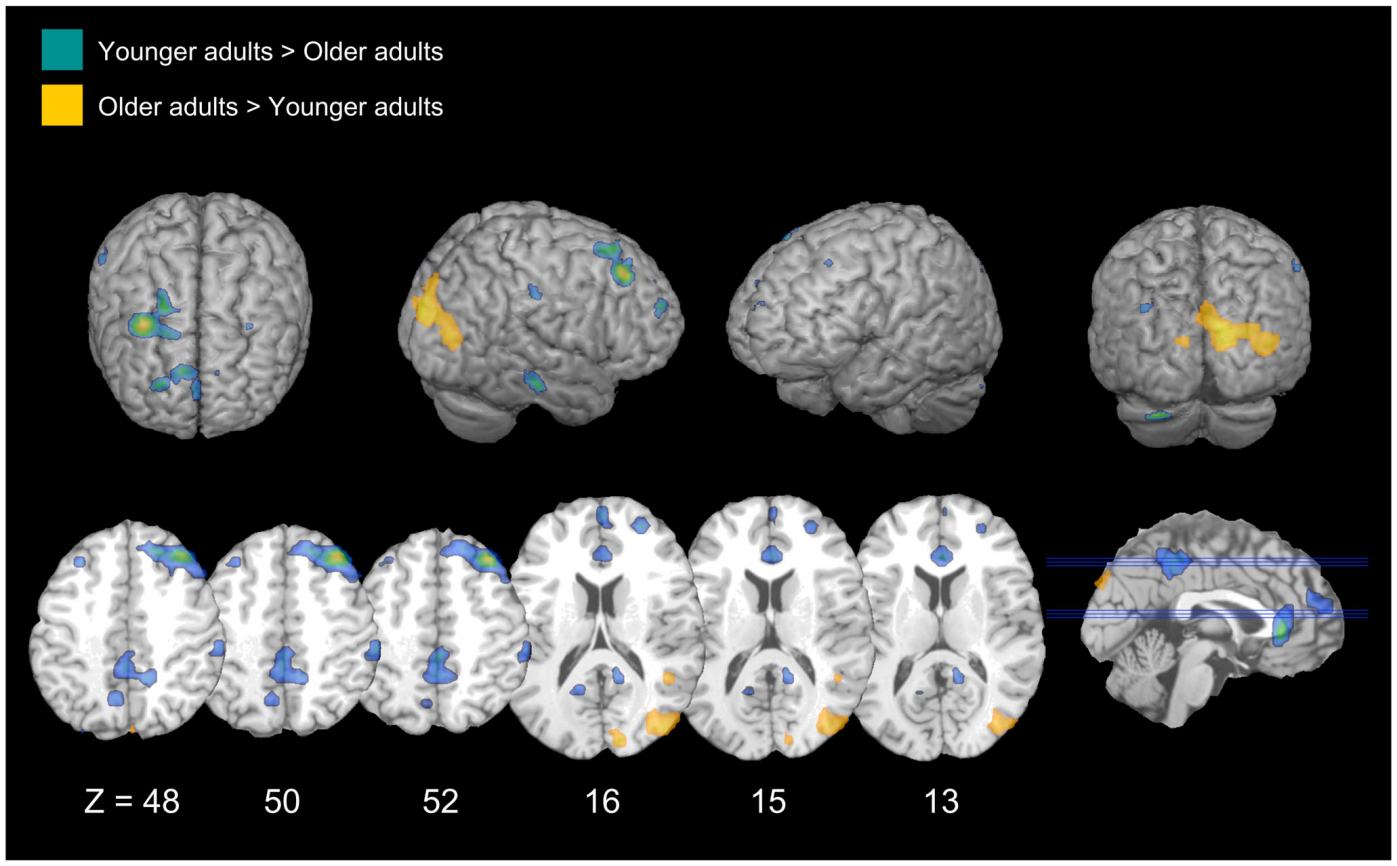
Age differences in successful intentional forgetting. Areas that are significantly more active for successful intentional forgetting than successful intentional remembering, and for which that difference is either greater in younger adults than older adults (blue), or greater in older adults than younger adults (yellow).

Consistent with the prediction that older adults would exhibit a reduction in inhibitory-related activity, younger adults recruited several right-lateralized regions to a greater extent than older adults during forgetting attempt and success. For attempt, these regions included the frontal, parietal, and temporal cortices. During forgetting success, younger adults recruited several regions within the right frontal and prefrontal cortices to a greater extent than older (see Table 4 & Figure 3).

**Table 4:** Intentional Forgetting.

	BA	H	Coordinates (T&T)			*t*	mm^3^
			*X*	*Y*	*Z*		
**Forgetting Attempt: TBF>TBR**							
**Younger Adults>Older Adults**							
Postcentral Gyrus	3, 1	R	63	−22	42	4.52	5377
Paracentral Lobule	1, 2	L	−59	−18	45	2.75	1317
	5	R/M	11	−29	53	4.26	8725
Precuneus	7	R	−19	−50	67	3.35	4280
Middle Temporal Gyrus	22	R	59	−35	5	2.79	2250
Inferior Parietal Lobe	40	R	56	−25	49	2.98	2689
**Older Adults>Younger Adults**							
Middle Temporal Gyrus	19	R	45	−81	21	3.51	1921
**Forgetting Success: TBF-F>TBR-R**							
**Younger Adults>Older Adults**							
Middle Frontal Gryus	10	R	26	57	14	2.93	1646
Superior Frontal Gyrus	8	R	37	33	43	4.13	4006
	9	R	11	50	21	3.13	1152
Paracentral Lobule	5	M	4	−33	49	2.60	5432
**Older Adults>Younger Adults**							
Middle Temporal Gyrus	19	R	45	−81	21	3.39	2250
Cuneus	19	R	11	−92	32	4.34	1646

BA = Brodmann’s Area; H = Hemisphere; *t* = statistical *t* value; T&T = Talairach and Tournix coordinates.

R = Right; L = Left; M = Midline.

#### Older adults>Younger adults

During forgetting attempts, older adults exhibited increased activation in the temporal cortex when compared to younger adults. During forgetting success, older adults exhibited increased activity in the right temporal and parietal regions, as compared to younger adults. (see Table 4 & Figure 3).

### Connectivity Analysis

The seed region for the PPI analysis was taken from the results of the intentional forgetting contrast in older adults (see Table S2). The right inferior parietal lobe was identified as the only region significantly more active for intentional forgetting than remembering that has also been previously implicated in inhibitory processing [10], [13]. Because the right inferior parietal lobe has been shown to be active during intentional forgetting across other fMRI studies, and was *also* significantly more active in older adults for intentional forgetting as compared to intentional remembering, the peak voxel from the older adults’ contrast was chosen as the seed region for the connectivity analysis.

When the time courses of the seed region in the right inferior parietal lobe (BA 40) was analyzed during TBF-forget trials, it was found to negatively covary with a cluster in the left MTL. Specifically, activity in BA 40 was negatively correlated with a cluster of voxels in the left hippocampus/parahippocampal gyrus (peak: −33, −35, −5; k = 1427 mm^3^). No significant negative interaction was found between the time course of voxels in the left inferior parietal lobe and any MTL region during TBR-recollection, TBF-recollection, or TBR-forget trial types. Thus, although activity in the right parietal lobe predicted a decrease in MTL activity during successful forgetting trials, it did not predict a similar decrease in activity during incidental forgetting, incidental recollection, or successful recollection. Previous connectivity analyses in younger adults failed to find a negative interaction between the parietal and hippocampal activity during intentional forgetting [13].

### Intentional (TBF-F>TBR-F) & Incidental forgetting (TBR-F>TBF-F)

#### Younger adults>Older adults

**Figure 4:**
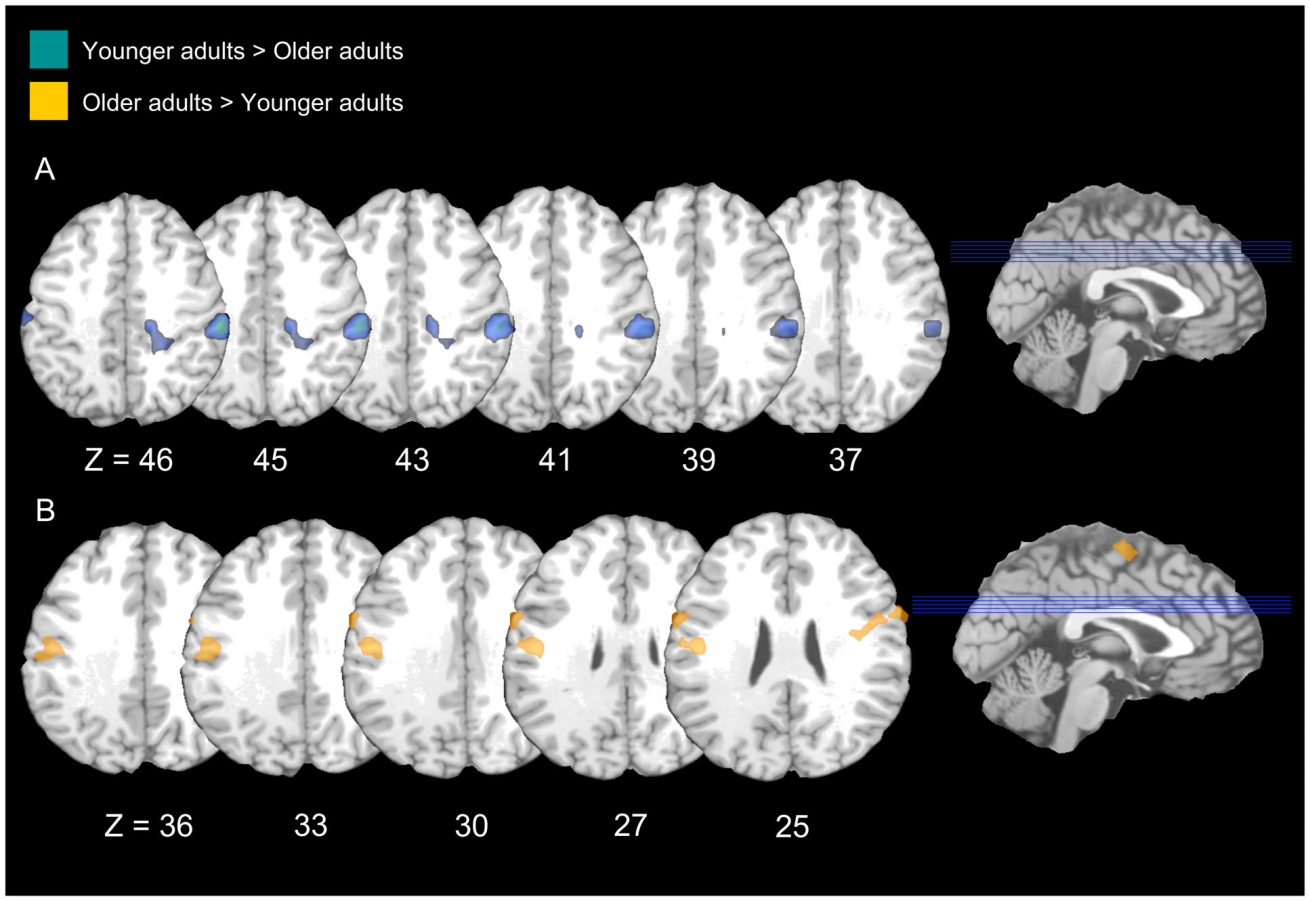
Age differences in intentional and incidental forgetting. A. Areas that are significantly more active for intentional forgetting than incidental remembering in younger compared to older adults. B. Areas that are significantly more active for incidental remembering than intentional remembering in older compared to younger adults.

Consistent with the hypothesis that the distinction between intentional and incidental forgetting would be reduced in older adults, younger adults, as compared to older adults, exhibited increased activation in the frontal and parietal cortices to a greater extent during intentional forgetting than incidental forgetting. Younger adults did not recruit any regions to a greater extent than older adults during incidental forgetting compared to intentional forgetting (see Table 5 & Figure 4).

**Table 5:** Intentional and Incidental Forgetting.

	BA	H	Coordinates (T&T)			*t*	mm^3^
			*X*	*Y*	*Z*		
**Intentional Forgetting: TBF-F>TBR-F**							
**Younger Adults>Older Adults**							
Postcentral Gyrus	2, 1	R	63	−25	45	5.95	4170
	2, 1	L	−67	−22	32	4.38	1427
Superior Parietal Lobe	5, 7	L	−26	−46	71	3.76	2414
	5, 7	R	19	−46	67	3.49	1921
Paracentral Lobule	7	R	11	−29	56	3.49	3073
**Older Adults>Younger Adults**							
**Incidental Forgetting: TBR-F>TBF-F**							
**Younger Adults>Older Adults**							
**Older Adults>Younger Adults**							
Middle Frontal Gyrus	11	L	−15	33	−15	2.84	2195
Inferior Frontal Gyrus	44	L	−41	13	12	2.51	3786
Postcentral Gyrus	1,2	L	−59	−19	35	3.86	6091
Precentral Gyrus/Frontal Eye Fields	6	R	48	2	16	3.23	2195
Postcentral Gyrus	2, 1	R	63	−25	45	5.95	8011
	2	L	−48	−32	60	2.42	2305
Paracentral Lobule	5	M	−7	−25	52	3.27	2963

BA = Brodmann’s Area; H = Hemisphere; *t* = statistical *t* value; T&T = Talairach and Tournix coordinates.

R = Right; L = Left; M = Midline.

#### Older adults>Younger adults

Older adults did not activate any regions to a greater extent than younger adults during intentional compared to incidental forgetting. They did, however, recruit several regions within the frontal cortex to a greater extent than younger adults during incidental compared to intentional forgetting (see Table 5 & Figure 4).

## Discussion

This study is the first to examine age differences in the neural correlates mediating cognitive control of memory during encoding using the DF paradigm. The analyses focused on age differences in both attempted and successful control of memory processes, specifically those of differential encoding and inhibition. Both processes have been shown to support intentional forgetting in younger adults [10], [13], [20] and both have been theorized to be impaired in older adults. While older adults did not exhibit the expected deficit in the directed forgetting effect, age-related deficits in d-prime and age-related increases in memory for TBF items indicates that older adults did experience a certain degree of memory impairment compared to younger adults. In addition, neuroimaging findings suggested differential neural recruitment between age groups. Specifically, results showed that encoding attempt was associated with greater neural recruitment of PFC and parietal cortex in older, compared to younger adults. Age-related increases in PFC activity, specifically in right inferior (BA 45) and left middle frontal (BA 10 & 11) gyri, were also associated with successful intentional encoding. Forgetting attempt was associated with age-related decreases in the right parietal lobe (BA 40). Older adults also exhibited decreased activity for successful intentional forgetting in several PFC regions, including right superior frontal gyrus (BA 8 & 9) and right middle frontal gyrus (BA 10); no age difference was observed in parietal cortex. Given that no PFC region showed successful intentional forgetting activity in older adults, connectivity analyses were performed between the right inferior parietal cortex and the MTL. Results showed a negative correlation between these two regions only during intentional forgetting. Finally, direct comparisons between intentional and incidental forgetting revealed that older adults, compared to younger adults, activated significantly less of the right parietal lobe (BA 5 & 7) during intentional forgetting as compared to incidental forgetting. However, older adults showed significantly more activity in left middle (BA 11) and inferior (BA 44) frontal gyri for incidental forgetting, as compared to intentional forgetting. Each finding is discussed in detail below.

### Intentional Encoding

In previous DF studies, encoding attempt is typically associated with activity in the left prefrontal, parietal, and early visual cortices [10], [13], [19]. While older adults exhibited a pattern of activity consistent with these previous results (see Table S1), they also exhibited age-related increases in activity in several regions within the PFC and parietal cortex, including the left middle frontal gyrus (BA 11), left superior frontal gyrus (BA 6), and left inferior parietal cortex (BA 40). Older adults also exhibited age-related decreases in activity in the right middle occipital gyrus (BA 18). These results support the posterior-to-anterior shift in aging (PASA) model, which posits that older adults compensate for age reductions in sensory processing regions by recruiting higher-order cognitive processes (i.e., PFC and parietal functioning) [25], [26].

Neural activity associated with encoding success was also relatively preserved in aging, with both younger and older adults exhibiting increased activation in the left inferior/middle (BA 47 & 10) and inferior (44 & 45) frontal gyri, left inferior temporal gyrus (BA 20), and the early visual cortex (BA 17) [13] (see Table S1). Activity in these regions is consistent with previous studies that examine encoding success and subsequent memory effects for verbal stimuli in both age groups [21], [64], [65]. These results suggest that older adults are able to recruit the typical encoding success network when executing intentional remembering within the item-method DF paradigm. Despite these similarities age differences did emerge. While older adults exhibited decreased activity in the superior frontal gyri (BA 6) and right occipital cortex (BA 18) they exhibited increased activity in a widespread network of frontal and temporal regions, including the right inferior frontal gyrus (BA 45), bilateral middle frontal gyri (BA 10 & 11), left superior frontal gyrus (BA 8), and left middle temporal gyri (BA 39). These results are consistent with a second often observed pattern in aging, the hemispheric asymmetry reduction in older adults (HAROLD) [29]. The model posits that cognitive functions that are typically lateralized in younger adults (i.e., left-lateralized encoding of verbal materials) recruit both hemispheres in older adults, particularly when the cognitive processes are executed successfully [29]. While age-related increases in bilateral activation have been labeled as representing both compensatory mechanisms [30], [49], [66] and dedifferentiation within neural substrates [33], [67], [68], the lack of behavioral differences in intentional remembering the current study favor the compensation hypothesis.

### Intentional Forgetting and Connectivity Analysis

While differential encoding represents the ability to enhance neural activation that supports later memory for relevant information, attentional inhibition represents one’s ability to actively suppress the encoding of irrelevant information. The current study found forgetting attempt to be associated with an age-related reduction in the neural recruitment of both right frontal and parietal regions compared to younger adults. To the extent that frontal and parietal regions support attempted forgetting through recruitment of inhibition mechanisms, our results suggest that older adults have difficulty recruiting inhibitory processes to the same extent as younger adults.

When considering forgetting success, older adults activated the right inferior parietal lobe (BA 40), but not the right superior or middle frontal gyrus (see Table S2). Furthermore, direct comparisons between age groups illustrate an age-related reduction in activation within right middle (BA 10) and superior (BA 8 & 9) frontal gyri, regions that were active during successful intentional forgetting in our group of young adults [13]. Thus, not only did older adults exhibit less right frontal activity than younger adults, they also did not differentially activate this region at all during successful intentional forgetting. Previous research has highlighted the involvement of both the right middle and superior PFC in inhibitory and control processes associated with memory, yet has not distinguished between processing across regions [13], [35], [36], [37]. While the right middle and superior PFC is associated with intentional forgetting success in both directed forgetting and think/no-think paradigms, increased activity in the these regions has also been directly linked to decreased activity in the hippocampus during successful intentional forgetting [13], [35], [36], [37], [41]. Considering these previous findings in young adults, the current results suggest that older adults are impaired in the recruitment of frontally mediated inhibitory processes. In fact, we did not find evidence for any frontal activity associated with successful intentional forgetting in older adults even when the threshold was reduced to *p<*0.01 uncorrected.

Despite the observed age-related reductions in frontally mediated activity associated with intentional forgetting, older adults did not exhibit a corresponding decrement in the directed forgetting effect. Results thus suggest that intentional forgetting can occur in the absence of PFC-mediated inhibition. Though the absence of prefrontal cortex activity was unexpected, a compensatory view of aging would suggest that, like many other cognitive processes, intact cognitive control in aging may be accomplished through a strategic shift in cognition or a shift in the neural regions that support a given task [25], [29]. While no region showed an age-related increase during successful intentional forgetting, older adults did not exhibit decreased activation of the right inferior parietal cortex. Considering the strong anatomical connections between the right PFC and right parietal cortex [69], [70], together with the fact that the parietal cortex is active during intentional forgetting across a wide range of ERP and fMRI studies [10], [13], [34], it appears that this region might be brought online as a way for older adults to perform the necessary inhibition of TBF items in lieu of reductions in prefrontal activity (specifically, the right superior frontal gyrus). These data, combined with that of younger adults, suggest that there are two separate inhibitory hubs, one in the PFC and one in the parietal cortex. Data further suggests that the parietal hub may come online in advanced age to inhibit the MTL, perhaps to compensate for processing deficits experienced in PFC regions. More research is necessary to identify under what circumstances this potential shift occurs.

In order to confirm this theory, we conducted a connectivity analysis between activity in the right inferior parietal cortex and the MTL (similar to that performed in younger adults [13]. Results showed that, in older adults, activity in the right inferior parietal lobe was negatively correlated with activity in the left MTL during successful intentional forgetting. Furthermore, this correlation was not observed during incidental forgetting, intentional remembering, or incidental remembering. Previous DF and TNT studies have interpreted such a negative correlation as support for an active inhibitory process underlying intentional forgetting [13], [35], [36], [37]. Using the same reasoning, a comparable conclusion could be drawn with regard to the parietal cortex activity in older adults. While it is unclear why older, but not younger, adults would utilize the parietal cortex for encoding inhibition, recent research showing that the parietal lobe modulates the relationship between the PFC and MTL during retrieval suppression [41] supports this interpretation.

Alternatively, the parietal lobe may support intentional forgetting through attentional withdrawal, with the negative correlation between parietal activity and that of MTL simply reflecting the relative engagement and disengagement of each region in intentional forgetting in older adults. Given the role of the parietal cortex in attention [71], older participants may use this region to re-focus their attention away from the TBF items, disengaging encoding operations. This diversion of attention, coupled with the observed differential encoding processes, may be enough to promote successful intentional forgetting. Future research is needed to distinguish between these two alternatives.

### Intentional and Incidental Forgetting

Finally, we were also interested in examining age differences in the relationship between incidental and intentional forgetting. With respect to regions showing greater activity for incidental than intentional forgetting, both younger and older adults activated the left inferior (BA 44 & 45) and superior (BA 6 & 8) frontal gyri [13] (see Table S3). Age comparisons found that older adults recruited significantly more activity during incidental than intentional forgetting in the left inferior (BA 44) and middle (BA 11) frontal gyrus compared to younger adults. Given evidence that these regions have been shown to support semantic processing [64], [65] and the retrieval of sensory details [59], [60], [72], results show that older adults engage in more encoding-related processing associated with the presentation of TBR items than TBF items, even when those TBR items are ultimately forgotten. These age comparisons reflect that incidentally forgotten items may have undergone *more* encoding attempt in older adults than younger adults. As such, our results converge on the finding that incidental forgetting is driven by encoding attempt that is not sufficient to result in successful memory at the time of retrieval.

Although the presence of two dissociable forgetting mechanisms, incidental and intentional forgetting, was clearly observed in younger adults [13], a similar finding was *not* observed in older adults. Older adults did not differentially recruit any inhibitory-related regions to a greater extent for intentional than incidental forgetting (see Table S3). Age comparisons confirmed that this lack of differentiation in aging, showing significantly reduced recruited of parietal cortex in older compared to younger adults during intentional forgetting, as compared to incidental forgetting.

Despite the lack of differentiation in neural activity between these two types of forgetting, older adults exhibited a significant behavioral difference between incidental and intentional, equivalent in magnitude to that observed in younger adults. In addition, the connectivity analyses indicate that activity in the right inferior parietal cortex is negatively coupled with MTL activity during intentional but not incidental forgetting. These results suggest that while there are minimal differences in the overall network recruited for intentional and incidental forgetting in aging, the role of the regions within the network may differ. Given the novelty of these results, more research is needed to explore the way in which these two findings can be reconciled. In particular, it will be important to investigate the neural correlates of intentional (and incidental) forgetting in a cohort of older adults who exhibit clear behavioral deficits in the directed forgetting paradigm.

## Conclusion

This study is the first to examine age differences in the neural correlates that mediate the cognitive control of memory encoding using the DF paradigm. The data extend previous findings by identifying possible mechanisms by which older adults execute both intentional encoding and intentional forgetting processes, that differ from those observed in younger adults. With regard to differential encoding, the results demonstrate a relatively intact intentional encoding network in older adults, when compared to younger adults. Where age differences do exist, the results are consistent with previous results of compensatory processing observed in incidental encoding tasks. However, age-related similarities in intentional forgetting activity were far less. Specifically, older adults showed a reduction in the recruitment of frontal inhibitory control regions while maintaining intact intentional forgetting related activity in the parietal cortex. Connectivity analyses indicate that this parietal activity negatively correlates with activity in the MTL during intentional forgetting. While only correlational, this suggests parietal cortex may play a role in active inhibition of encoding processes in older adults who show reductions in frontally-mediated inhibition. Finally, we provide evidence that older adults do not show a strong dissociation between the neural regions that support intentional and incidental forgetting. Taken together, the current study suggests that neural processes that support cognitive control of memory through inhibition differ between young and older adults.

## Supporting Information

Figure S1 **Seed region for connectivity analysis.** Location of the parietal cluster from which the peak voxel (in pink) was taken for the PPI connectivity analysis in older adults (extracted from TBF-Forget>TBR-Recollect contrast).
(TIF)

Table S1 **Intentional Remembering in Older Adults.**
(TIF)

Table S2 **Intentional Forgetting in Older Adults.**
(TIF)

Table S3 **Intentional & Incidental Forgetting in Older Adults.**
(TIF)
