# Supplementary figures and images for: The Cognitive Control of Memory: Age Differences in the Neural Correlates of Successful Remembering and Intentional Forgetting
Source: PLoS One. 2014 Jan 24;9(1):e87010. doi: 10.1371/journal.pone.0087010 (PMC3901730; doi:10.1371/journal.pone.0087010)

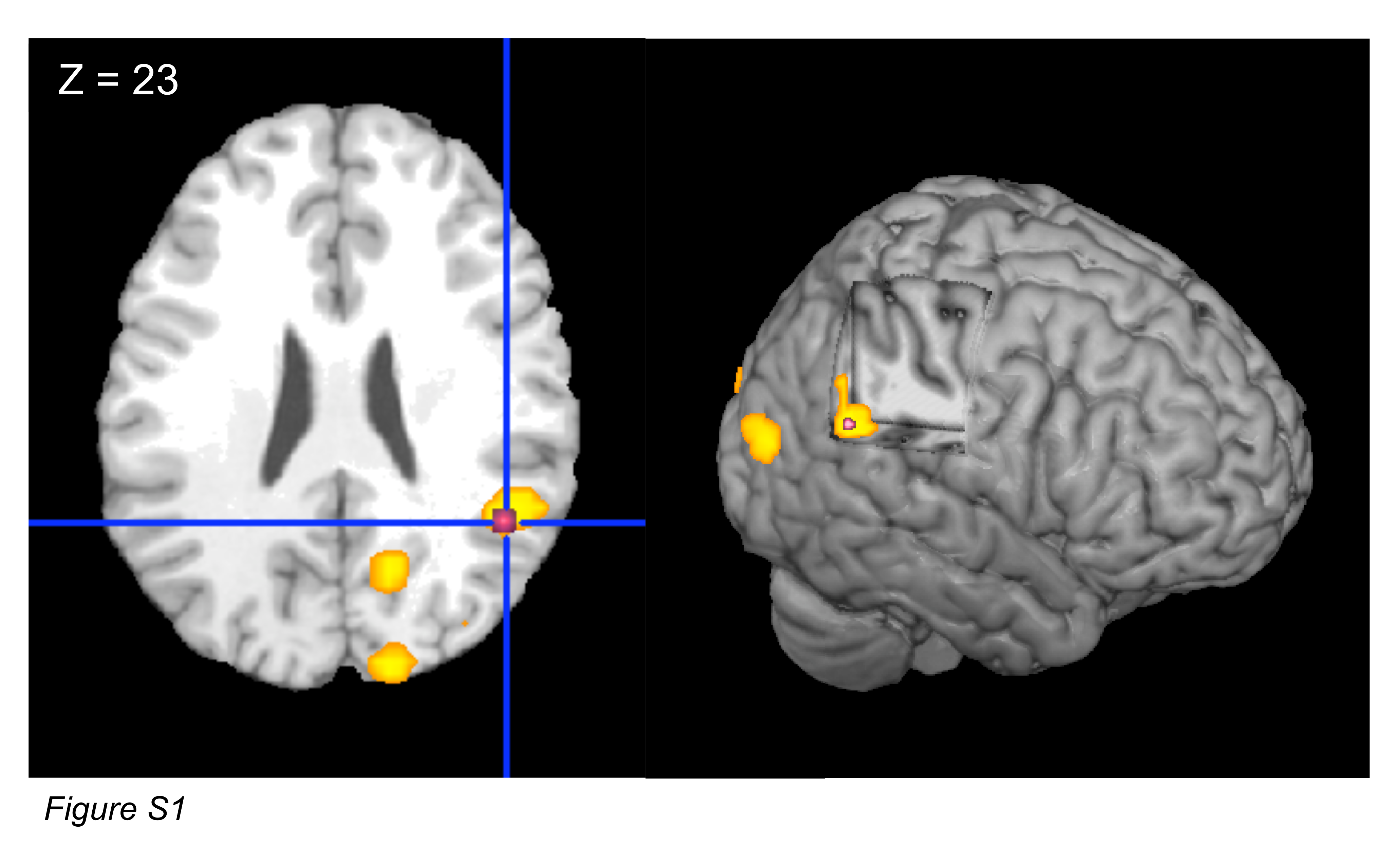

Supplement: Figure S1 — Seed region for connectivity analysis. Location of the parietal cluster from which the peak voxel (in pink) was taken for the PPI connectivity analysis in older adults (extracted from TBF-Forget>TBR-Recollect contrast). (TIF) [file pone.0087010.s001.tif]
